# Grounding Intuitive Physics in Perceptual Experience

**DOI:** 10.3390/jintelligence11100187

**Published:** 2023-09-26

**Authors:** Michele Vicovaro

**Affiliations:** Department of General Psychology, University of Padua, 35122 Padua, Italy; michele.vicovaro@unipd.it

**Keywords:** intuitive physics, naïve physics, perceptual experience, misconception, heuristic, Bayesian cognitive model, phenomenology, impetus

## Abstract

This review article explores the foundation of laypeople’s understanding of the physical world rooted in perceptual experience. Beginning with a concise historical overview of the study of intuitive physics, the article presents the hypothesis that laypeople possess accurate internalized representations of physical laws. A key aspect of this hypothesis is the contention that correct representations of physical laws emerge in ecological experimental conditions, where the scenario being examined resembles everyday life experiences. The article critically examines empirical evidence both supporting and challenging this claim, revealing that despite everyday-life-like conditions, fundamental misconceptions often persist. Many of these misconceptions can be attributed to a domain-general heuristic that arises from the overgeneralization of perceptual-motor experiences with physical objects. To conclude, the article delves into ongoing controversies and highlights promising future avenues in the field of intuitive physics, including action–judgment dissociations, insights from developmental psychology, and computational models integrating artificial intelligence.

## 1. Introduction

Scientific physics relies on precise measurement instruments, mathematical tools, and the minds of skilled scientists. These resources are unavailable to those who lack formal training yet seek to comprehend the underlying rules governing the behavior of physical objects in their environment. Intuitive physics, also known as naïve physics, refers to the understanding of the physical world held by individuals without formal instruction in physics (Anderson 1983; Bertamini and Casati 2009; Bianchi and Savardi 2014; DiSessa 1993; Kubricht et al. 2017; McCloskey 1983; Vicovaro 2021). Here, I focus on the relationship between perceptual experience and the intuitive understanding of the physical world among laypeople. This review article can be useful to the non-specialist as a broad up-to-date introduction to the topic of intuitive physics. It can also be of interest to the specialist reader for the following reasons. There is a widespread agreement that intuitive physics is rooted in perceptual experience. However, while it was previously generally accepted that perceptual experience leads to an inaccurate representation of the physical world, a recently developed theory inspired by Bayesian cognitive modeling posits that perceptual experience provides a truthful source of information about the physical world. A core argument in the theoretical perspective based on Bayesian cognitive models is that many of the systematic errors that had been documented in the early studies on intuitive physics were related to the abstract and unrealistic nature of the task. According to this perspective, because intuitive physics is grounded on everyday life perceptual experience, accurate intuitive knowledge of physical phenomena would emerge in ecological conditions, that is, when realistic rather than abstract and simplified stimuli are used, when dynamic simulations are presented instead of static depictions, and when the task is presented from a first-person action-oriented perspective rather than from a third-person allocentric perspective.

After a brief historical introduction to the study of intuitive physics (Section 2), the present work aims to fill a gap in the literature by providing a careful review of the empirical evidence in support or against this claim (Section 3 and Section 4). Additionally, in Section 5, I propose that a domain-general heuristic based on the overgeneralization of perceptual experience provides a fruitful theoretical framework for the interpretation of many misconceptions about physical phenomena. Lastly, Section 6 highlights some open controversies and future directions in research on intuitive physics.

## 2. A Brief Historical Overview of the Study of Intuitive Physics

Albert Michotte’s ground breaking work on the perception of causality (Michotte [1946] 1963) can be considered the precursor to psychological studies in the field of intuitive physics. In Michotte’s experiments, participants were presented with simple two-dimensional animations featuring two horizontally aligned squares. In these animations, one square (*A*) would initiate movement towards the initially stationary square (*B*) at a certain point in time. Upon contact, *B* would start moving in the same direction as *A* had been moving, while *A* came to a halt. Under specific conditions, observers reported an overwhelming visual impression that *A* had caused the motion of *B*, known as the launching effect. Michotte extensively manipulated various parameters of the animation, such as the relative speeds and trajectories of *A* and *B*, and even introduced delays during their contact, to explore how such manipulations influenced observers’ impressions. Occasionally, Michotte also referenced Newtonian laws of collisions to demonstrate that the launching effect is governed by inherent laws of the visual system, distinct from physical laws. However, as pointed out by Runeson (1983), Michotte’s focus was primarily on the visual impressions of causality, disregarding other intriguing aspects of intuitive physics related to collisions. These aspects include the overall perceived plausibility of different collisions and the perception of the dynamic properties of the objects involved, such as their relative masses. 

The first investigations into what later on has been called intuitive physics were conducted by Paolo Bozzi, who examined the visual perception of pendulum motion and motion along inclined planes (for a recent reprint of a translation of Bozzi’s work, see Bressan and Gaudiano 2019). In his study on pendulum motion (Bozzi 1958), he discovered that the period of oscillation that appears most natural to observers is slower than the physically accurate period. This finding was partially supported by a later study by Pittenger (1990). Similarly, Bozzi (1959) found that the motion of an object along an inclined plane perceived as most natural by observers does not align with the physically correct accelerated motion. Instead, it involves initial acceleration followed by a period of constant velocity, which corresponds to a uniform velocity motion at a perceptual level (Bozzi 1989). Furthermore, Bozzi noted a positive correlation between the size (implied mass) of the object and the velocity perceived as most natural for descent. However, from a physical standpoint, velocity should be independent of mass if we assume negligible friction. According to Bozzi (1989), the visual perception of physical events is guided by intrinsic laws of the visual system that operate independently of physical laws. This viewpoint bears resemblance to Michotte’s ([1946] 1963) theoretical perspective on the perception of causality.

Michotte’s contributions significantly shaped research in the field of the visual perception of physical events. Notably, studies in this area differ from conventional intuitive physics, in that they emphasize visual perception over reasoning processes and conscious predictions. These vision-focused studies have demonstrated that basic two-dimensional visual displays can evoke a variety of visual impressions of physical phenomena. These include bouncing (Vicovaro et al. 2023), braking (Levelt 1962), bursting (White and Milne 1999), generative transmission (White 2015), penetration (White and Milne 2003), pulling (White and Milne 1997), and shattering (Hubbard and Ruppel 2013). Furthermore, observers have the ability to extrapolate the dynamic properties of physical objects involved in mechanical events (Runeson 1983), such as their masses (Runeson and Vedeler 1993; Runeson et al. 2000; Sanborn et al. 2013) and elasticity (De Sà Teixeira et al. 2014; Paulun and Fleming 2020; Vicovaro and Burigana 2016; Warren et al. 1987). When physical constraints are violated, it can lead to the perception of animated, self-generated motion (Parovel 2023). Additionally, there is evidence suggesting that certain relatively complex relational properties of physical events are processed early by the visual system, preceding the involvement of higher-level cognitive processes (Hafri and Firestone 2021; Little and Firestone 2021; Wong et al. 2023).

The field of intuitive physics started capturing the attention of cognitive psychologists towards the late 1970s and early 1980s. During this time, a series of studies brought forth striking disparities between the fundamental laws of mechanics, such as Newton’s first and second laws, and people’s performance in simple tasks that assessed their intuitive understanding of these laws (Anderson 1983; Caramazza et al. 1981; Kaiser et al. 1985a, 1985b, 1986a, 1986b; McCloskey et al. 1980, 1983; McCloskey and Kohl 1983; Shanon 1976; Yates et al. 1988). This wave of empirical studies gave rise to the development of what is perhaps the most well-known theory in the field of intuitive physics, known as the naïve impetus theory (McCloskey 1983); see also Kaiser et al. (1986a). According to the naïve impetus theory, laypeople’s conceptualization of object motion bears resemblance to the medieval impetus theory, which posits that an object moves when an impetus is imparted to it, gradually dissipating and causing a gradual deceleration of the object itself (McCloskey 1983). As highlighted by Hubbard (2022) and others, impetus transmission serves as a reasonable heuristic in a world marked by the pervasive presence of friction, although it contradicts the Newtonian concept of inertia, wherein an object would move at a constant speed in the absence of external forces in an idealized frictionless world.

Alongside the endeavors of cognitive psychologists, researchers in the field of science education also made significant contributions in the 1980s by examining the underlying causes of students’ difficulties in learning physics. It became evident that students’ misconceptions regarding basic physical phenomena are resistant to change and lead to systematic distortions in their understanding of the concepts taught in physics courses (Champagne et al. 1980; Clement 1982; DiSessa 1993; Halloun and Hestenes 1985). Even fourth-year university physics students, as observed by Sequeira and Leite (1991), could still harbor misconceptions about fundamental laws governing gravitational motion. To address these challenges, it became increasingly clear that explicit discussions and the refutation of students’ misconceptions is necessary to prevent the distortion and accommodation of new physics concepts to the existing biased knowledge held by students (Carey 1989; McDermott 1991). By actively confronting and correcting these misconceptions, physics teachers could enhance the effectiveness of their instruction and facilitate meaningful learning experiences for their students.

During the 1990s, there was a temporary decline in the interest of cognitive scientists in intuitive physics. One notable exception was a heated debate surrounding the role of direct perception and heuristics in visual judgments of the relative masses of objects involved in collisions (Gilden and Proffitt 1994; Runeson and Vedeler 1993). Additionally, studies emerged during this period that explored how animations and expertise influenced participants’ performance in intuitive physics tasks (Hecht and Proffitt 1995; Kaiser et al. 1992; Proffitt et al. 1990). A renewed interest in the field of intuitive physics emerged in the 2000s, marked by a series of important studies on various topics. These studies examined laypeople’s intuitive understanding of reflections in mirrors (Bertamini et al. 2003a, 2003b; Bertamini et al. 2010; Croucher et al. 2002; Hecht et al. 2005; Lawson and Bertamini 2006; Lawson et al. 2007; Savardi et al. 2010), the motion of projectiles (Hecht and Bertamini 2000; Huber and Krist 2004; Krist 2000; Oberle et al. 2005; Shaffer and McBeath 2005; Twardy and Bingham 2002), the motion of objects along inclined planes (Rohrer 2002, 2003), and collisions (White 2007). These studies built upon the research tradition by primarily focusing on the psychological foundations of the disparities between intuitive and scientific physics.

The 2010s marked the emergence of a new approach to studying intuitive physics, which was based on Bayesian cognitive modeling (Battaglia et al. 2013; Kubricht et al. 2017; Sanborn et al. 2013; Ullman et al. 2017). This approach involves comparing participants’ performance in intuitive physics tasks with predictions generated by Bayesian models. These models combine prior probability distributions based on Newtonian laws with current perceptual information that is characterized by stochastic noise. The underlying idea is that the brain stores the correct representations of physical laws as prior knowledge, which are then utilized in the simulation process, similar to the physics engines used in interactive video games (Ullman et al. 2017). These Bayesian models account for potential discrepancies between participants’ performance and predictions based on physical laws by attributing them to perceptual noise (Battaglia et al. 2013; Sanborn et al. 2013) or the use of computational shortcuts aimed at reducing processing costs. For example, the simulation process may selectively focus on certain elements while neglecting others (Bass et al. 2021; Ullman et al. 2017).

In summary, there has been a shift in the study of intuitive physics since the 2010s. Earlier research primarily focused on highlighting the discrepancies between laypeople’s intuitive understanding of physics and the formal scientific laws. However, from the 2010s onwards, there has been an attempt to reconcile intuitive physics with scientific physics. Bayesian cognitive models have become the mainstream approach in studying intuitive physics, as evident in recent publications (Bass et al. 2021; Fischer 2021; Fischer and Mahon 2021; Lau and Brady 2020; Neupärtl et al. 2021). Nevertheless, it is worth noting that the idea that intuitive physics is influenced by heuristics and biases remains a topic of discussion and exploration (Bianchi and Savardi 2014; Hecht 2015; Hubbard 2022; Ludwin-Peery et al. 2021a; Vicovaro et al. 2021; White 2009).

## 3. A Review of Studies on How Realism Augments Performance in Intuitive Physics Tasks

One of the key concepts in the Bayesian cognitive simulation models of intuitive physics, as proposed by Battaglia et al. (2013), is that simulation mechanisms have evolved to facilitate an effective interaction with the physical environment. According to this perspective, laypeople internalize accurate representations of physical laws through perceptual experience. These simulation mechanisms are optimized for real-life or real-life-like scenarios and may not be as effective in abstract reasoning tasks. As a result, accurate performance in intuitive physics tasks is expected to emerge only when perceptual experience is involved. For example, individuals are expected to be more accurate in judging the naturalness of an animation compared to making predictions in static abstract conditions. Fischer and Mahon (2021) have suggested that many errors in physical reasoning problems can be explained by the mismatch between how these problems are framed and the native format of inferences generated by cognitive physical engines. These inferences are grounded in a first-person, action-oriented perspective. Mistakes can occur when applying this perspective to the abstract and allocentric perspective involved in many intuitive physics tasks. In summary, Bayesian cognitive simulation models propose that simulation mechanisms optimized for real-life scenarios contribute to accurate performance in intuitive physics tasks that involve perceptual experience. However, challenges arise when applying these mechanisms to abstract reasoning tasks or tasks that require a shift in the perspective from first-person action-oriented to abstract and allocentric. According to this perspective, a systematic difference should emerge between intuitive physics tasks that involve real-life like scenarios, which should be associated with high performance accuracy, and intuitive physics tasks that involve abstract scenarios, which should be associated with poor performance.

Bayesian models have been employed to predict participants’ performance across various physical scenarios, characterized by realistic simulations of everyday life physical events and by the use of dynamic simulations rather than statistic depictions. These include the stability of simulated 3D block towers (Battaglia et al. 2013; Hamrick et al. 2011), collision dynamics (Gerstenberg et al. 2012; Lau and Brady 2020; Sanborn et al. 2013; Smith and Vul 2013), liquid dynamics (Bates et al. 2015), ballistic motion (Bass et al. 2021; Smith et al. 2013, 2018), and causal judgments (Gerstenberg et al. 2012; Sanborn et al. 2013). Notably, participants consistently demonstrated performance aligned with the predictions generated by Bayesian simulation models, suggesting an internalized and accurate representation of relevant physical laws. 

Even prior to the emergence of Bayesian cognitive simulation models, empirical studies had already presented evidence that highlighted the influence of stimulus realism and real-life scenarios on the performance of laypeople in intuitive physics tasks. For example, Kaiser et al. (1986b) conducted a study comparing participants’ trajectory predictions in familiar and unfamiliar situations. They presented participants with scenarios involving a ball exiting from a curved tube and water exiting from a curved hose. While these situations were physically identical, participants performed more accurately in the familiar version of the task compared to in the unfamiliar version. This suggests that familiarity with the scenario improves performance in intuitive physics tasks. Similarly, Masin et al. (2014) found that participants had correct intuitive knowledge of the equilibrium of a lever but poor intuitive knowledge of the equilibrium of hydraulic pressures, despite the formal equivalence of the underlying physical laws. They argued that the accurate intuitive knowledge of lever equilibrium may be based on perceptual-motor experiences with familiar activities, such as playing on a seesaw. However, there is no corresponding everyday life experience that would provide a basis for an intuitive understanding of the hydraulic pressure equilibrium.

Other studies have suggested that the presence of animation, or motion, can positively impact the accuracy of participants’ performance in intuitive physics tasks. Shanon (1976) found that participants who falsely believed that objects fall downward at a uniform speed were able to correctly judge uniform acceleration as the more natural motion, when presented with videos of falling objects, but see Vicovaro et al. (2019). Kaiser et al. (1985a) found that participants were more likely to make incorrect interpretations based on impetus transmission when presented with static versions of a motion scenario, compared to with animated versions of the same scenario. In a similar vein, Kaiser et al. (1992) reported that participants’ responses were more consistent with the predictions from Newtonian physics when making visual judgments of the naturalness of simulated motion compared to making cognitive predictions of unseen motion. Hecht et al. (2005) found that using dynamic simulation and real-life tasks, as opposed to abstract paper-and-pencil tasks, reduced errors in participants’ understanding of the physical behavior of mirrors. However, it is important to note that even in ecological experimental situations, systematic errors persisted (Bertamini et al. 2003b, 2010; Bianchi and Savardi 2012; Bianchi et al. 2015).

Not only visually perceived motion, but also imagined motion and imagined action, can sometimes lead to performance improvement in intuitive physics tasks. Huber and Krist (2004) conducted a study where participants were presented with a scenario involving a sphere falling off a horizontal surface positioned at varying heights from the ground. In a dynamic version of the task in which participants were able to use mental imagery to simulate the parabolic falling motion of the sphere, they were able to accurately predict the time-to-contact based on the surface height alone. In contrast, in a static version of the task where mental imagery was not used by participants (as shown based on eye movement analysis), the participants’ estimated time-to-contact was also influenced (incorrectly) by the horizontal speed of the sphere. Schwartz and Black (1999) found that predictions about the surface orientation of a liquid inside a glass were more accurate when participants had to imagine actively tilting the glass compared to when they had to make predictions based on an abstract conceptualization of the physical situation. Corneli and Vicovaro (2007) reported that participants made incorrect predictions when asked to reason about friction between an object and a surface in abstract terms. However, when participants were asked to imagine pushing the object along the surface, they made correct predictions. These studies suggest that mental imagery and imagined actions can facilitate intuitive reasoning about physical situations, potentially providing individuals with a more embodied and experiential understanding of the underlying physical principles.

In scenarios involving a collision where an object *A*, initially in motion, approaches an object *B* initially at rest, research indicates that participants often tend to focus on and report the force exerted by object *A* on object *B*. However, they frequently neglect or underestimate the force exerted by object *B* on object *A*, thereby deviating from the principles of Newton’s third law (White 2007). However, this bias has been shown to be reduced, although not completely eliminated, when participants are presented with simulated collisions involving realistic 3D spheres that appear to be made of different simulated materials (Vicovaro 2012, 2018). In these studies, the use of realistic 3D simulations resulted in more accurate ratings of the perceived naturalness of collisions, which were also more consistent with the predictions from Newtonian laws, compared to when abstract immaterial shapes were used (Vicovaro and Burigana 2014).

In summary, the literature that I have reviewed thus far suggests a general positive correlation between the realism of scenarios and participants’ accuracy in intuitive physics tasks. However, as I will discuss in the following section, a significant body of research indicates that errors often occur even when realistic stimuli and scenarios are employed.

## 4. Exploring Studies Challenging the Notion That Realism Eliminates Fundamental Misconceptions

In a paper-and-pencil task conducted by McCloskey et al. (1983), participants were asked to predict the trajectory of a ball released by a carrier moving along a horizontal path. Surprisingly, about half of the participants incorrectly anticipated that the ball would fall straight to the ground in a vertical line, disregarding the parabolic trajectory resulting from the initial horizontal motion. This misconception, known as the straight-down belief, seems to extend to real-life situations as well. In a subsequent experiment, participants were instructed to walk along a straight path while holding a ball and release it to hit a target on the floor. Strikingly, approximately half of the participants mistakenly released the ball directly above the target, causing it to miss (developmental studies on the straight-down belief are discussed by Kaiser et al. 1985b; Krist 2000). McCloskey et al. (1983) also presented participants with videos and animations of a projectile released by a moving carrier. Surprisingly, once again, about half of the participants reported that the projectile fell in a straight vertical trajectory rather than a parabolic path. The authors attributed this misperception to the fact that the presence of the moving carrier produced a distortion in the perceived trajectory of the target, speculating that this perceptual experience contributes to the formation of the straight-down belief; see also Kaiser et al. (1985b).

In a study involving a ball-throwing scenario (Hecht and Bertamini 2000), participants were asked to predict the point in its trajectory where a ball thrown by a human thrower would reach maximum speed. Despite the fact that the ball would begin to decelerate immediately after leaving the thrower’s hand according to the laws of physics, participants inaccurately predicted that the maximum speed would occur at approximately one-third of the parabolic trajectory. This suggests a belief that the ball continued to accelerate even after its release. This bias was observed not only in a static version of the task but also when participants viewed virtual animations of throwing actions. Vicovaro et al. (2012, 2014) employed realistic throwing animations generated through motion capture techniques. They found that observers were insensitive to significant manipulations of both the thrower’s motion and the projectile’s motion.

In a study by Rohrer (2002), participants were presented with two curved wooden ramps, both initially descending concavely. However, one of the ramps had an additional concave curvature (dip ramp), while the other had an additional convex curvature (hill ramp). The participants were instructed to imagine two identical marbles rolling down each ramp and predict which marble would have the highest speed at a specified target position. Neglecting the effects of friction, which were negligible in this context, the speed of a marble at any point on the ramp would be directly proportional to the square root of its vertical distance from the starting point of descent. Importantly, the slope of the ramp at that particular point would not impact the marble’s speed. Through a series of multiple-choice questions, Rohrer (2002) found that participants falsely believed in a proportional relationship between speed and slope, known as the slope–speed belief. This belief suggests that the speed of an object on an incline increases with the slope of the incline at that point. In a follow-up study, Rohrer (2003) presented participants with animations of a ball falling down a curved ramp and asked them to assess the naturalness of different motion patterns. The results demonstrated that the majority of participants perceived animations depicting a simulated ball moving according to the slope-speed pattern as more natural compared to animations showing the ball moving according to the physically correct motion pattern. Therefore, the slope-speed belief is not limited to static scenarios but also extends to dynamic situations.

In numerous paper-and-pencil tests, it has been observed that laypeople commonly overestimate the influence of mass on the speed of objects falling vertically downward or along an inclined plane due to gravitational attraction. They tend to believe that heavier objects fall faster than lighter objects (Champagne et al. 1980; Halloun and Hestenes 1985; Karpp and Anderson 1997; Oberle et al. 2005; Proffitt et al. 1990; Shanon 1976; Whitaker 1983). Even university physics students have demonstrated this belief (Sequeira and Leite 1991). This misconception, known as the mass–speed belief (Rohrer 2002), contradicts Newtonian physics, which states that all objects fall toward the center of gravitational attraction with the same uniform acceleration in a vacuum (i.e., in the absence of friction). Vicovaro (2014) found that this misconception persists even when participants are asked to predict the unseen falling speed of real objects that they can manipulate. To explore how gravitational motion is perceived, Vicovaro et al. (2019) presented participants with real-scale simulations of wooden (i.e., heavy) or polystyrene (i.e., light) spheres falling from a height of approximately two meters. The participants were then asked to rate the naturalness of each simulated fall. The results revealed that physically impossible high-speed values were judged as natural for wooden spheres, while physically impossible low-speed values were judged as natural for polystyrene spheres. Additionally, simulated motions that were physically incorrect but featured uniform speed were perceived to be approximately as natural as uniformly accelerated motions. In a second experiment, Vicovaro et al. (2019) demonstrated that the mass–speed belief also influenced participants’ estimates of the time it took for simulated spheres, with partially obscured trajectories, to make contact with a surface (see also Vicovaro et al. 2021). It is important to note that if the effects of air resistance are considered, the mass–speed belief cannot be simply dismissed as an error. Due to the influence of air resistance, heavier objects tend to fall slightly faster than lighter ones (Oberle et al. 2005; Vicovaro et al. 2021). However, Vicovaro et al. (2021) showed that mass still affects the perceived naturalness of falling acceleration, even when the simulated effects of air resistance are included in the animation. This suggests that even in highly realistic scenarios, laypeople are susceptible to the mass–speed belief.

Ludwin-Peery et al. (2021a) conducted a study in which participants were shown videos depicting realistic simulations of two spheres falling. These spheres rolled down ramps, platforms, and steps or were set in motion by a series of dominoes (for an example: OSF). Each video played for 2 s before being interrupted, and participants were then asked to predict which sphere would touch the ground first. The spheres differed not only in their falling paths (such as rolling on different ramps or encountering steps) but also in other factors, including speed, distance covered at the moment the animation was paused, and the presence of obstacles. Surprisingly, the results indicated that participants performed below the chance level, suggesting that they were unable to accurately reconstruct the relative timing of the motion of the two spheres.

**Figure 1:**
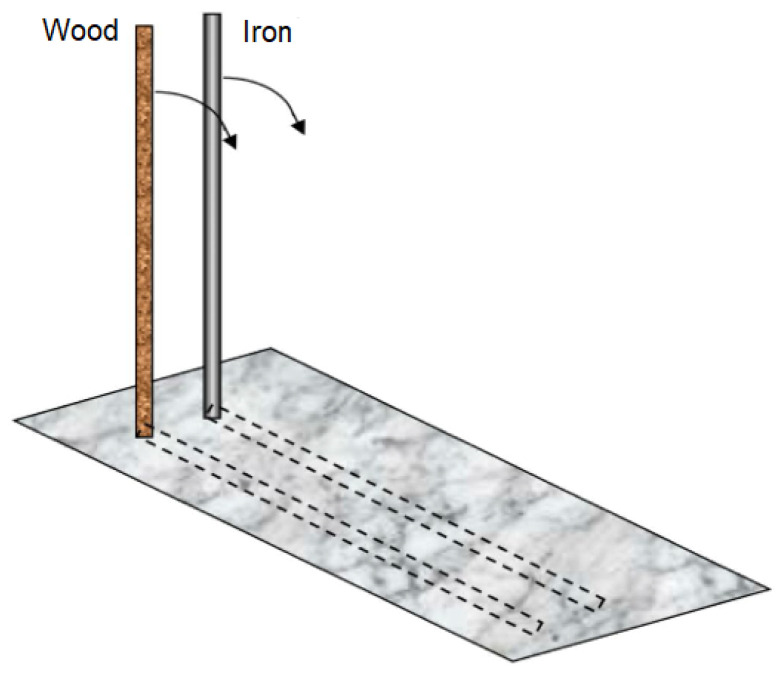
Hypothetical wooden and iron rods in the task used by Hecht (2015). Arrows indicate the hypothetical motion direction, dash lines indicate the hypothetical landing positions. The figure is reproduced and adapted with the permission of the author.

Hecht (2015) conducted a study using static pictures of vertical rods to investigate participants’ predictions about the falling time and balancing difficulty of these rods. The rods could vary in three dimensions: weight, length, and the position of an additional weight attached at the top or bottom of each rod (see Figure 1, for an example of a scenario characterized by rods differing in weight). Participants were tasked with predicting which rod would fall quicker to the ground if both were slightly tipped over simultaneously, as well as which rod would be easier to balance. The latter task aimed to tap into participants’ perceptual-motor experiences related to balancing or hefting objects. From a physical perspective, the falling time of a rod depends solely on the distance of its barycenter from the ground (i.e., the base of the rod). Balancing difficulty should be inversely proportional to the falling time, as a shorter falling time provides less time to balance the rod. However, less than half of the participants provided correct responses in the abstract version of the task, and even the more concrete version centered on motor experience did not yield improved performance. In line with the mass–speed belief, slightly over 40% of participants incorrectly predicted that the heavier rod would fall faster than the lighter rod. Perceptual-motor knowledge fared even worse, with slightly over 70% of participants incorrectly believing that the heavier rod was harder to balance, despite the balancing difficulty being the same for both rods.

Croucher et al. (2002) conducted research to explore people’s intuitive understanding of mirror physics, commonly referred to as naïve optics. They found that a significant percentage of individuals held the misconception that when a person walks parallel to a flat mirror, they can see their reflection in the mirror before being directly in front of it (referred to as an early error). This misconception consistently emerged in both paper-and-pencil tests and real-life scenarios, with the errors being more pronounced in real-life situations than in the tests. In another study focusing on laypeople’s understanding of mirror image size, Lawson and Bertamini (2006) asked participants to estimate the size of their face’s mirror reflection. A substantial overestimation error was observed, as participants incorrectly believed that the mirror projection of their face was approximately the same size as their actual face, whereas it should be half the size (regardless of the participant’s distance from the mirror). Lawson et al. (2007) further demonstrated that this overestimation error extended to arbitrary objects, such as bamboo sticks of varying lengths, and also encompassed images projected on windows. Interestingly, providing perceptual feedback through the observation of actual projections on mirrors or windows only led to a modest increase in accuracy for the projection size estimation task (Lawson and Bertamini 2006; Lawson et al. 2007). In the context of naïve optics, Bertamini et al. (2003a) revealed that people hold incorrect representations of what is visible in mirrors. For example, when participants were presented with Velazquez’s painting “The Toilet of Venus” (also known as “The Rokeby Venus”), they erroneously reported that Venus was seeing herself in the mirror, although this would be physically impossible. This phenomenon, referred to as the Venus effect, extends beyond paintings to include photographs and realistic everyday scenarios. It appears to be linked to individuals’ insensitivity to the fact that the portion of space visible in a mirror varies based on the observer’s position relative to the mirror (Bertamini et al. 2010; Bianchi and Savardi 2012).

## 5. The Role of Heuristics in Laypeople’s Understanding of the Physical World

Despite claims that physical laws are internalized through perceptual experience and that the use of realistic frames of reference is crucial for accurate performance in intuitive physics tasks (Fischer and Mahon 2021; Smith et al. 2018), the literature reviewed in the previous section indicates that even in realistic and familiar physical scenarios, fundamental mistakes can occur. Some errors appear to be directly linked to perceptual experience. For example, the straight-down belief seems to be influenced by the fact that an object released by a moving carrier appears to fall straight down due to the presence of the moving carrier (McCloskey et al. 1983). In the field of naïve optics, Lawson et al. (2007) proposed that people tend to significantly overestimate the size of mirror images because they do not perceive them as images on the mirror surface, but rather they perceive them as virtual copies beyond the mirror of the corresponding real objects. These virtual copies are perceived to be the same size as the original images due to a size constancy mechanism. However, there is a lack of other instances of direct perceptual errors, suggesting that misleading visual experiences cannot fully explain the wide range of errors observed in the domain of intuitive physics.

Many misconceptions regarding the motion of objects seem to stem from an *overgeneralization* of our perceptual-motor experiences during interactions with physical objects. To illustrate this, let us consider the perception of kicking a ball from our perspective. Initially, the action is planned in our mind, generating a sense of agency and willingness. In contrast, the ball is perceived as passive and inanimate. When the foot makes contact with the ball, we feel a certain resistance exerted by the ball, which varies based on factors, such as its mass. If the kick is sufficiently strong and no external factors, like wind, interfere, the ball moves in the anticipated direction, its speed being directly proportional to the force of the kick and inversely proportional to the ball’s resistance. Subsequently, the ball gradually decelerates until it comes to a stop. Scholars have observed that laypeople interpret mechanical interactions between physical objects in a manner consistent with this phenomenological framework (DiSessa 1993; Hubbard 2022; Klaassen 2005; McCloskey 1983; White 2009). For example, when presented with a collision scenario involving a moving object *A* colliding with a stationary object *B*, individuals tend to identify *A* as the agent and *B* as the passive object, or the patient. They believe that *A* imparts a directional impetus to *B*, which in turn resists the transmission of this impetus. Consequently, after the collision, the speed of *B* is believed to increase as the impetus (i.e., force) from *A* increases and to decrease as *B*’s resistance increases. As the impetus gradually dissipates, *B* is expected to decelerate until it eventually halts. Thus, there exists a strong parallel between how laypeople perceive first-person interactions with physical objects and how they conceptualize mechanical interactions involving inanimate objects. In other words, it appears that perceptual-motor experiences during personal interactions serve as an interpretative framework for comprehending mechanical interactions between physical, non-living objects (i.e., an overgeneralization of perceptual-motor experiences occurs).

Relying on phenomenal experience leads laypeople to believe that when an object applies or experiences a force in a particular direction, it will move in that direction with a speed directly proportional to the force (referred to as force as a mover, as discussed by DiSessa 1993)^1^. This principle, when (over)generalized, can potentially account for systematic biases, such as the mass–speed belief and the slope–speed belief. Regarding the mass–speed belief, Vicovaro et al. (2021) observed that when an object is held in hand, it exerts a downward force on the hand, with the magnitude of this force directly proportional to the object’s mass. Consequently, a heavy steel ball exerts a much stronger downward force than a light polystyrene ball. If the perceived force is used as an indicator of the object’s falling speed, it naturally leads to the idea that the heavy steel ball falls much faster than the light polystyrene ball. Similarly, Rohrer (2002) noted that the force exerted by an object in the direction of an incline is directly proportional to the slope of the incline at that point. Once again, if force is utilized as a predictor of speed, the observer would believe in a direct proportional relationship between the slope and speed, which was indeed observed in Rohrer’s studies.

It is essential to highlight that the aforementioned errors do not stem from a complete lack of understanding of physical principles. Instead, they arise from the overgeneralization of principles that hold true within the context of motor interactions between an individual and an inanimate object, leading to invalid solutions in other contexts (as also discussed by Hecht 2001). Overgeneralization errors have also been documented in the field of naïve optics. Bianchi and Savardi (2014) highlighted that many errors made by observers when predicting what is visible in a mirror can be attributed to the inappropriate generalization of their most typical perceptual experiences. For instance, observers often rely on their typical experience of standing in front of a mirror to predict what would be visible when they are positioned to the side of the mirror (see also Bianchi and Savardi 2012). Similarly, some participants mistakenly believe that when they walk parallel to a mirror surface, the mirror image will initially appear on the opposite side of the mirror and move in the opposite direction of their walking. These misconceptions likely arise from the erroneous generalization of the common perceptual experience that the mirror image behaves contrary to the observer (see also Bianchi et al. 2015).

Overgeneralization errors can be viewed as a type of heuristic. Since the influential work of Tversky and Kahneman (1974), the term heuristic has been widely employed in the field of probabilistic and causal reasoning. It refers to simplified reasoning strategies that individuals use instead of more complex normative probability laws. The adoption of heuristics is typically attributed to the limited capacities of the cognitive system and the computational demands of probability laws. For instance, let us consider the assessment of a potential causal relationship between a source event *X* (e.g., a specific medical treatment) and an outcome event *Y* (e.g., recovery from a specific medical condition). Normatively, determining the existence or absence of a causal link between *X* and *Y* requires applying Bayes’ theorem, which involves combining the relative frequencies of different events (e.g., *X* & *Y*, ¬*X* & *Y*, *X* & ¬*Y*). However, research has consistently shown that laypeople tend to rely on simplified heuristics in such cases. One common heuristic is to count the relative frequency of the outcome event *Y*, disregarding the frequencies of ¬*X* and *Y*, as well as *X* and ¬*Y*. Although this heuristic may yield satisfactory results in certain contexts, it is not a universally valid strategy. Consequently, its usage can lead to a significant overestimation of the strength of the causal link between the source and outcome events, known as a causality bias (Blanco et al. 2013).

Overgeneralization errors in intuitive physics and biases in probabilistic reasoning exhibit some common characteristics. Firstly, both are reasonably accurate in specific contexts. Secondly, laypeople tend to apply them even when they cannot yield valid solutions. Thirdly, they allow for significant computational resource savings. As noted by Hecht (2015), as long as heuristics provide reasonably accurate solutions for the majority of scenarios encountered in everyday life, there is no compelling reason to abandon them in favor of more accurate but computationally expensive representations of reality. Bertamini and Casati (2009) emphasized that heuristics can be viewed as a by-product of efficient adaptation to the environment in certain cases. For example, research has shown that waitresses, compared to other populations, are more prone to errors when judging how the surface orientation of liquid in a glass changes when the glass is tilted (Hecht and Proffitt 1995). The errors made by waitresses appear to arise from an excessive focus on the frame of reference provided by the glass, which can be attributed to the significance of this reference frame in their daily tasks as waitresses.

In this article, our primary focus centers on heuristics concerning object motion, proposing that they stem from the overgeneralization of perceptual-motor experiences. However, it is important to acknowledge that there exist other types of heuristics that are predominantly visual in nature. For instance, Ludwin-Peery et al. (2021a) found that participants rely on a relatively simple visual resemblance heuristic to determine the most probable final state of an unstable tower of blocks following its collapse. Moreover, a recent study conducted by Liu et al. (2023) revealed that visual inputs associated with object-related geometry, particularly the centroid, are sufficient for accurate predictions regarding object stability.

From a theoretical perspective, the development of heuristics in the field of intuitive physics involves a certain degree of knowledge systematization and generalization. The underlying idea is that perceptual experience is utilized to construct knowledge structures that encompass various physical phenomena. In his influential theoretical work, DiSessa (1993) termed these knowledge structures *phenomenological primitives* or *p-prims*. Without delving into details, there exists a debate regarding the size and interconnectedness of these knowledge structures. On one end, McCloskey (1983) proposed that individuals’ intuitions about motion resemble the structure of a non-Newtonian scientific theory, such as the Medieval impetus theory, wherein general universal principles coexist with specific cases and exceptions. On the other end, DiSessa (1993) suggested that people’s intuitions about the physical world are organized into relatively small and weakly interconnected knowledge structures, limited in scope to a few specific situations. According to this viewpoint, intuitive physics lacks the significant systematic nature found in theoretical science. Yates et al.’s (1988) prototypes theory suggests that the resolution of physics problems relies on the activation of a prototype representing the specific physical scenario. These prototypes are acquired through perceptual-motor experiences with the physical world and stored in long-term memory. Unlike the other theories discussed, the prototypes theory does not propose a systematic organization of knowledge. Instead, prototypes are considered isolated fragments of knowledge of which activation is influenced by various contextual factors, including the perceptual salience of different aspects of a given physical scenario as determined by the task at hand.

In concluding this section, I would like to highlight that the characterization of intuitive physics through heuristics is not a novel concept. As mentioned earlier, numerous empirical findings have been illuminated through this lens. Nevertheless, a comprehensive theoretical framework encompassing these heuristics remains elusive. In this contribution, I aim to propose the existence of a domain-general heuristic—specifically, an overgeneralization of perceptual experience—that can elucidate a wide range of systematic prediction errors observed in domains, such as collisions, throwing, vertical falling motion, and mirrors.

It is important to acknowledge that the type of perceptual experience evoked during an intuitive physics task may vary based on specific stimulus features (Yates et al. 1988). For instance, as discussed in Section 3, participants may disregard the force exerted by a stationary object on a moving object when presented with a collision between two abstract shapes (White 2007). However, participants seem to take this backward force into account when exposed to realistic simulations, particularly when the stationary object appears to be made of a dense material, like iron (Vicovaro 2018). This latter scenario could potentially elicit perceptual-motor experiences of rebounding against a massive object, such as a large stone or a wall. Furthermore, studies conducted within the theoretical framework of Bayesian cognitive models (Section 4) provide compelling evidence that participants’ judgments and predictions align with Newtonian laws under specific circumstances. While the validity of internalizing Newtonian constraints may not hold universally, certain physical laws do seem to be internalized by individuals. Deciphering the factors that determine when and why the accurate internalized knowledge of physical principles supersedes the use of heuristics presents an ongoing and significant challenge for theories of intuitive physics.

## 6. Current Controversies and Future Directions

Over the past decade, Bayesian simulation models have gained significant popularity. These models are based on the principle that perceptual experience serves as a reliable source of information regarding physical phenomena. The closer a scenario resembles everyday life experiences, the more accurate participants’ judgments and predictions are expected to be. Fischer and Mahon (2021) express this idea by stating that scenarios presented from an egocentric perspective are more likely to yield accurate predictions compared to those presented from an allocentric perspective (p. 459). However, a considerable body of literature contradicts this claim by demonstrating that systematic errors can occur even in ecologically valid scenarios (Section 4). Noteworthy examples include laypeople consistently overestimating the influence of mass on the falling speed of objects. This tendency persists not only when participants engage in abstract paper-and-pencil tasks but also when they make predictions about the unseen falling motion of real objects that they can manipulate (Vicovaro 2014). Moreover, fundamental misunderstandings about mirror reflections emerge when participants are exposed to both schematic drawings and real mirrors (Bertamini et al. 2010; Bianchi and Savardi 2012; Croucher et al. 2002). Furthermore, it is important to note that the allocentric perspective does not necessarily hinder performance in intuitive physics tasks (Huber and Krist 2004; Kaiser et al. 1992). Consequently, the notion that a realistic first-person perspective is the key determinant of response accuracy does not seem to be well-supported by empirical evidence.

In addition to the previous point, it is worth highlighting that Ludwin-Peery et al. (2021a) have recently challenged the psychological plausibility of Bayesian cognitive models of intuitive physics through the empirical testing of three core predictions derived from these models. These predictions are as follows: (1) the mental simulation should effectively consider all relevant elements of the physical scenarios that contribute to the outcome; (2) the temporal order of events should be accurately represented and maintained throughout the simulation; (3) the outcome of the mental simulation process should align with the principles of normative probability theory. Regarding the first prediction, the authors found that participants exhibited a poor ability to detect changes in the number and shape of blocks in a tower when asked to predict its most likely future state following a collapse caused by poor balancing. In relation to the second prediction, participants were asked to predict which of two balls would touch the ground first after rolling down a series of ramps and obstacles. Surprisingly, they tended to reverse the exact order of arrival, indicating that the mind struggles to perform a faithful simulation of mechanical events that takes the precise time course into account. Lastly, concerning the third prediction, participants demonstrated a conjunction fallacy when making predictions about the unseen potential outcome of a collision between two flying balls. Specifically, they judged the simultaneous occurrence of two events (e.g., ball *X* being hit by ball *Y* and ball *X* touching the grass) as more likely than the occurrence of a single event (e.g., ball *X* touching the grass) alone. As a result, these findings contradict the predictions made by Bayesian models, revealing that probabilistic reasoning in the context of simple mechanical events appears to violate the normative laws of probability (see also Ludwin-Peery et al. 2021b for additional instances of the conjunction fallacy in the context of simple mechanical events).

Bass et al. (2021) put forth an argument challenging the assumption that a Bayesian cognitive simulation model necessarily aligns with predictions based on physical laws. They suggest that individuals might employ a strategy of partial cognitive simulation to conserve computational resources. This approach entails conducting a detailed physical simulation solely for the most relevant element of a scenario, while disregarding less significant elements, even if they might hold importance from a physical standpoint. Drawing an analogy from physics simulation engines utilized in action video games, these neglected elements are referred to as sleeping elements (Ullman et al. 2017). Their behavior is simulated only when they become salient within the scenario. According to this perspective, the perceptual realism of an animation depends on the physical plausibility of the behavior exhibited by the salient objects, whereas realism is not expected for non-salient elements. Thus, the differentiation between active and sleeping elements allows for the preservation of perceptual realism while conserving valuable computational resources. However, two unresolved issues arise from this viewpoint. Firstly, determining the criteria by which the cognitive system identifies which elements should undergo Newtonian simulation and which ones can be considered sleeping elements remain a challenge. Secondly, as previously discussed in Section 4, instances exist where significant deviations from physical predictions are accepted even for obviously salient objects, such as the trajectory of a thrown ball, the elements that are visible in a mirror, or the speed of a falling object.

Another intriguing debate concerns the role of intuitive physics in everyday motor interactions with physical objects. Some researchers propose that the cognitive system and the motor-action-oriented system employ distinct representations of physical phenomena (Fischer and Mahon 2021; Zago and Lacquaniti 2005). They suggest that while the cognitive system may rely on sub-optimal heuristic representations of physical laws, motor actions are driven by accurate internalized representations of these laws and constraints. These action-oriented representations are cognitively impenetrable, meaning they cannot generate correct responses in tasks unrelated to action. For example, in relation to the common misbelief that heavier objects fall faster than lighter objects, it has been shown that this erroneous belief influences cognitive predictions of unseen falling motion (Vicovaro 2014), the perceived naturalness of visually perceived falling motion (Vicovaro et al. 2019, 2021), and the imagined motion of falling objects (Vicovaro et al. 2021). However, Lacquaniti and Maioli (1989) demonstrated that this belief had no impact on the timing of the manual interception of vertically falling objects. Participants were able to catch a falling sphere at the correct moment, regardless of its mass. In general, empirical evidence suggests that accurate internalized representations of Earth’s gravity play a crucial role in guiding both the manual interception of vertically falling objects (McIntyre et al. 2001; Zago et al. 2008) and the ocular pursuit of targets on parabolic trajectories (Jörges and López-Moliner 2019), although some authors have questioned the necessity of these representations (Baurès et al. 2007; Zhao and Warren 2015).

Supporting the dissociation between action and cognition in intuitive physics, Smith et al. (2018) conducted a study involving a pendulum scenario. Participants were asked to draw the path that the pendulum’s bob would take if the cord was cut. They were presented either with static depictions of a pendulum scenario or with animations stopped at the moment of cord cutting. Marked discrepancies between participants’ predictions and the theoretically correct responses emerged in both conditions. However, when a more action-oriented version of the task was introduced, where participants adjusted the position of a bucket to catch the ball after the cord was cut or cut the cord to make the ball land in a bucket, their responses aligned substantially with physics. By analyzing individual response patterns from participants who took part in both the static and action-oriented conditions, it became evident that participants relied on different knowledge systems. Another study by Neupärtl et al. (2021) explored an immersive virtual reality scenario where participants propelled real pucks varying in mass towards targets at different distances. In this setting, participants relied on a correct Newtonian model. However, in a more abstract version of the task presented on a monitor with interactions using key presses, participants used a simplified heuristic model. In a study conducted by Krist et al. (1993), it was found that children demonstrated an understanding of the Velocity = *D*/√*H* rule when manually propelling a tennis ball along a horizontal surface, to make the ball land on a target positioned on the ground at a vertical height *H* from the surface and at a horizontal distance *D* from the edge of the surface. However, when asked to predict the unseen velocity of the ball, they failed to consider the influence of height. The same disconnect between motor and cognitive abilities was observed in other areas, such as time, space, and velocity integration (Huber et al. 2003; Wilkening and Martin 2004). At a more theoretical level, it is worth underlining that the definition of action itself remains an open question. For instance, Zago and Lacquaniti (2005), Neupärtl et al. (2021), and Krist et al. (1993) conceptualize action as motor interactions with physical objects, while Smith et al. (2018) had participants engage with abstract virtual representations of objects. Consequently, the boundaries of the domain to which this hypothetical action-oriented knowledge system applies are nuanced.

The hypothesis that motor interactions with physical objects are guided by internalized representations of physical laws and constraints is undeniably intriguing. It offers an explanation for the efficacy of these interactions, even when cognitive processes rely on heuristics. In simpler terms, one does not necessarily need precise cognitive and perceptual representations of physical phenomena to successfully adapt to the environment, as long as actions are guided by accurate internalized representations of physical laws. Nonetheless, due to the limited number of empirical studies examining motor interactions with physical objects, further investigation is necessary to delve deeper into this hypothesis. It is worth mentioning that a dissociation between action and cognition was not observed in a developmental study that employed a slingshot throw paradigm (Krist 2003), as well as in a study involving children aged 6 to 12 and adults who were tasked with releasing a ball onto a target located on the ground while they moved horizontally (Krist 2000). Moreover, McCloskey and Kohl (1983) found that a curvilinear impetus heuristic could account for both cognitive predictions and motor interactions involving moving objects.

This review primarily focused on studies involving adult participants, but it is worth noting that there is a vast and valuable field of research dedicated to the development of intuitive physics in infancy and childhood. Across a wide range of physical scenarios, studies have demonstrated that children gradually acquire the ability to predict outcomes by integrating multiple physical variables using additive, multiplicative, and averaging rules (Anderson and Wilkening 2014; Hill et al. 2014; Jäger and Wilkening 2001; Léoni and Mullet 1993; Wilkening 1981; Wilkening and Martin 2004; Vicovaro 2021). Some noteworthy studies on children’s intuitive understanding of elementary mechanics are discussed in the remainder of this review. Regarding the understanding of vertical falling motion, research indicates that the belief that heavy objects fall faster than light objects emerges as early as 5–6 years of age and remains consistent throughout primary school (Hast 2014; Hast and Howe 2012, 2015). In contrast, concerning motion along an inclined plane, the belief that heavy objects roll down faster than light objects tends to emerge between the second and fourth year of primary school, while first-year children hold the opposite belief (Hast 2014; Hast and Howe 2017). The relative saliency of vertical and horizontal motion components appears to play a significant role in this gradual transition (Hast 2016). Interestingly, when children were presented with videos of real balls rolling down an inclined transparent tube, the physically correct motion pattern, where the light and heavy balls have the same speed, was perceived as more accurate compared to incorrect motion patterns, such as a higher speed for either the light or heavy ball (Hast and Howe 2017). Similar results were found for vertical falling motion (Hast and Howe 2015). Overall, these findings suggest a potential dissociation between children’s implicit and explicit knowledge, although caution should be exercised when drawing conclusions. To further examine this claim, it is important to note that these studies involved presenting participants with two videos simultaneously—one showing the falling motion of a heavy sphere and the other showing the falling motion of a light sphere. The videos were either presented at natural speed (equal speed condition) or with one video slowed down to half its natural speed (different speed condition). This manipulation resulted in a substantial speed difference between the heavy and light objects, with one of the objects moving inconsistently with the effects of Earth’s gravitational acceleration. Given this consideration, it is possible that a scenario where a heavy object falls faster than a light object (or vice versa) may be perceived as more natural than an equal speed scenario if a subtler speed manipulation is employed.

In a noteworthy developmental study conducted by Kaiser et al. (1986a), participants in the age range of 5–12 years and adults were presented with spiral, C-shaped, or straight tubes placed on the ground. They were asked to predict the trajectory that a ball rolling inside the tube would follow upon exiting. All participants predicted a straight trajectory when the ball exited from the straight tube. However, a divergence in responses emerged for the spiral and C-shaped tubes across different age groups. The majority of children aged 10 to 12 tended to incorrectly predict a curvilinear trajectory, while adults and surprisingly, the youngest children (5 to 6 years old), tended to predict a physically correct straight trajectory. In other words, performance accuracy varied with age following a U-shaped function. The authors suggested that this phenomenon might be attributed to children initially relying on visual experiences to predict the ball’s trajectory, which led them to find the correct solution. However, as their understanding developed, they gradually learned that objects tend to continue moving in the same direction unless external forces intervene. Overgeneralizing this reasonable experience-based heuristic led them to falsely believe that the ball would follow a curvilinear trajectory upon exiting the tube.

A new frontier in research on children’s intuitive understanding of the physical world involves the construction of neural networks that mimic children’s knowledge of physics. Notably, studies have shown that neural networks trained with thousands of videos of real physical motion can learn to predict the future positions of moving objects, even through unsupervised learning processes (Ehrhardt et al. 2018). Recently, Piloto et al. (2022) developed a neural network trained with 28 h of observation of 3D scenes depicting simulated physical events. Following this training, the neural network demonstrated the ability to predict object behavior based on principles, such as continuity, object persistency, solidity, unchangeableness, and directional inertia. Importantly, these principles constitute the fundamental building blocks of infants’ understanding of the physical environment (Baillargeon 2002). Beyond these core concepts, it would be intriguing to construct neural networks trained through perceptual-motor interactions with the physical world rather than the mere observation of physical events. Testing the generalization of knowledge acquired through perceptual-motor training using new physical scenarios could provide further insights into the processes underlying the construction of intuitive representations of the physical world.

## 7. Summary

Comprehending how the cognitive system constructs a representation of the physical world is a valuable yet challenging endeavor. It advances our theoretical grasp of human representation of the external world and holds practical implications, particularly for enhancing physics education.

This review centered on the hypothesis that intuitive physics relies on simulations rooted in internalized physical laws, yielding precise solutions in real-life scenarios. I examined empirical evidence both supporting and refuting this notion. Notably, misconceptions often persist, even in realistic situations involving perceptual judgment or active interactions with tangible objects. A domain-general heuristic, stemming from the overgeneralization of perceptual-motor experience with physical objects, was proposed as a potential explanation for many such misconceptions. However, the precise conditions under which accurate task-specific knowledge may override this general heuristic remains a contentious subject necessitating future investigation.

Finally, I underscored the significance of action–judgment dissociations and developmental models based on neural networks as promising research avenues within the realm of intuitive physics.

## Data Availability

Not applicable.

## Note

1 The relationship between object speed and the resistance offered by an object to an applied force is thought to be inversely proportional. This notion helps shed light on why children often hold the belief that when a fixed force is applied to two objects initially at rest on a horizontal plane, the lighter object will move faster than the heavier one, and the smallest object will move faster than the larger one (Hast and Howe 2012).
